# Facets of Psychopathy, Intelligence, and Aggressive Antisocial Behaviors in Young Violent Offenders

**DOI:** 10.3389/fpsyg.2019.00984

**Published:** 2019-05-14

**Authors:** Fernando Renee González Moraga, Danilo Garcia, Eva Billstedt, Märta Wallinius

**Affiliations:** ^1^Regional Forensic Psychiatric Clinic, Växjö, Sweden; ^2^Department of Clinical Sciences Lund, Child and Adolescent Psychiatry, Lund University, Lund, Sweden; ^3^Centre for Ethics, Law and Mental Health, Institute of Neuroscience and Physiology, The Sahlgrenska Academy, University of Gothenburg, Gothenburg, Sweden; ^4^Blekinge Center for Competence, Region Blekinge, Karlskrona, Sweden; ^5^Department of Psychology, University of Gothenburg, Gothenburg, Sweden; ^6^Gillberg Neuropsychiatry Centre, Institute of Neuroscience and Physiology, The Sahlgrenska Academy, University of Gothenburg, Gothenburg, Sweden

**Keywords:** psychopathy, aggression, violence, intelligence, antisocial behavior, offenders, prison

## Abstract

Psychopathy continues to be a challenge in forensic contexts, and evidence of its association with destructive behaviors, such as aggressive antisocial behaviors, is extensive. However, the potential role of intelligence as moderator of the well-established association between psychopathy and aggressive antisocial behaviors has largely been neglected, despite intelligence having been independently related to both concepts. Increased knowledge of whether intelligence is relevant to this association is needed because of its possible implications on the assessment and treatment of individuals with psychopathic traits and aggressive antisocial behaviors. This study aimed to investigate the association between psychopathic traits, aggressive antisocial behaviors, and intelligence in young violent offenders and to test whether intelligence moderates the relationship between psychopathic traits and aggressive antisocial behaviors. Participants were 269 male violent offenders aged 18–25 years, assessed on the Psychopathy Checklist-Revised (PCL-R), the Life History of Aggression (LHA), and the General Ability Index from the Wechsler Adult Intelligence Scale, 3rd edition. Associations were tested with Spearman’s rho, and moderation analysis was performed through ordinary least squares regressions. The PCL-R four-facet structure was used for the analyses. We found a positive association between psychopathic traits and aggressive antisocial behaviors, and a small negative association between the Affective PCL-R facet and intelligence. In the moderation analyses, a small yet statistically significant moderation effect of intelligence on the association between the Interpersonal facet and LHA total scores was demonstrated. However, the amount of variance in the LHA total score explained by the model was very small: 2.9%. We suggest that intelligence, however, important for rehabilitation strategies and everyday functioning, is not necessarily pertinent to understanding aggressive antisocial behaviors in young offenders with psychopathic traits.

## Introduction

People with highly psychopathic traits are a resource-intensive group in the criminal justice system, and they often return to crime despite extensive rehabilitative efforts (Rice and Harris, 2013). Clinicians meet and treat these people in forensic psychiatry and other healthcare situations where they pose major challenges, possibly because those with highly psychopathic traits often do not complete their treatment. Even small reductions in aggressive antisocial behaviors require substantial resources, thus imposing substantial economic cost to society (DeLisi et al., 2017). Currently, no specific treatment has been found effective in reducing the general criminality or aggressive antisocial behaviors of this population (Hecht et al., 2018).

Psychopathy is considered the most important clinical construct in the criminal justice system (Hare, 1999; Logan and Hare, 2008) due to its implications for sentencing, placement, treatment planning, and risk assessment (Hare, 1996). People with highly psychopathic traits are often described as social predators without conscience but with a sense of grandiosity, lack of remorse or shame, low empathy, high impulsivity, low anxiety, and high levels of thrill-seeking and criminal behaviors (Cleckley, 1950; Hare, 1985, 1999; Babiak and Hare, 2006). Taken together as a construct, these interpersonal, affective, and behavioral characteristics make this a unique group for the criminal justice and healthcare systems to deal with (Hare, 2003; Shaw and Porter, 2012). The prevalence of psychopathy is estimated at about 1% in the general population (Neumann and Hare, 2008; Coid et al., 2009a) and 15 to 25% in prison populations (Hare, 1999), with lower rates in Europe than in North America (Coid et al., 2009b). Despite being a minority, people with highly psychopathic traits commit more than 50% of the most serious crimes (Hare et al., 1993; Hare, 1999) and display high rates of criminal versatility (Kosson et al., 1990; Shaw and Porter, 2012). There is compelling evidence that psychopathy is related to aggressive antisocial behaviors including proactive aggression (Cornell et al., 1996; Raine et al., 2006; Reidy et al., 2011; Wallinius, 2012; Pardini et al., 2014), and psychopathy remains an important predictor of both violent and non-violent recidivism (Hart et al., 1988; Hare and Neumann, 2008; Pedersen et al., 2010; Wallinius et al., 2012; Dhingra and Boduszek, 2013; Sánchez et al., 2014; Harris et al., 2017). However, there has been substantial critique to the notion that psychopathy predicts criminal recidivism, since items measuring criminal/antisocial behaviors are incorporated in the PCL-R and its derivatives. In fact, studies have demonstrated that the predictive ability of the PCL-R is mainly carried by the Antisocial facet (Walters et al., 2008, 2011; Wallinius et al., 2012), which is constituted by items measuring antisocial behaviors. In line with this, scientists and clinicians have argued for psychopathy to be reformulated in accordance with the original descriptions by Cleckley (1950), focusing on interpersonal and affective components as a core feature of psychopathy (Boduszek et al., 2016; Debowska et al., 2018), and several alternative measures of psychopathy have thus been constructed. Recently, a study applying network analysis to map out the core features of psychopathy in large samples of offenders assessed with the PCL-R showed cross-cultural differences with callousness/lack of empathy as the single most central PCL-R item for North American samples, while irresponsibility and parasitic lifestyle were the most important PCL-R items in a Dutch sample (Verschuere et al., 2018). The debate continues about whether antisocial behaviors are a core component of the construct (Hare and Neumann, 2010; Neumann et al., 2015) or a consequence (Skeem and Cooke, 2010a,b) and what features are integral to the construct (Lilienfeld et al., 2016).

One factor previously related to both aggressive antisocial behaviors and psychopathy is intelligence. Research has shown that offenders, including violent offenders, tend to score lower on measures of intelligence than non-offenders, especially in terms of verbal abilities (Beaver et al., 2013; Schwartz et al., 2015; Kavish et al., 2018). However, early descriptions of people with highly psychopathic traits indicated a positive relationship with intelligence, i.e., good, or even superior, intelligence (Cleckley, 1950). Furthermore, illustrating the complexity of the relation, it has been suggested that high intelligence in those with highly psychopathic traits is associated with early onset of aggressive antisocial behaviors and more problematic behaviors within and outside institutions (Johansson and Kerr, 2005). Thus, high intelligence may not be a hallmark of psychopathy, as proposed by Cleckley (1950), but could rather be a factor that increases the destructive potential of people with psychopathic traits.

Although previous research provided evidence of some relation between psychopathy and intelligence, the role of intelligence as a possible moderator of the well-established relationship between psychopathy and aggressive antisocial behaviors is largely unknown. The only known published study of this demonstrated that intelligence and psychopathy interact, with higher levels of psychopathy and intelligence linked to higher levels of crime among young offenders (Hampton et al., 2014). However, this link has not been replicated in other groups. If intelligence is considered a factor in assessing the risk of violence, it could have important implications for developing forensic treatment interventions and in formulating care efforts for offenders with highly psychopathic traits. Moreover, research that considers the possible moderation of intelligence on the association between psychopathy and aggressive antisocial behaviors needs to consider possibly different effects for different facets of psychopathy, since previous research indicates that psychopathy as described in the PCL-R is more heterogeneous than unitary (Yildirim and Derksen, 2015).

The aim of the current study was to investigate whether the relationship between psychopathic traits and aggressive antisocial behaviors is moderated by intelligence in a well-described, nationally representative cohort of young violent offenders in Sweden, with the following specific research questions: (1) Which associations can be found between psychopathic traits, aggressive antisocial behaviors, and intelligence? and (2) Does intelligence moderate the relationship between psychopathic traits and aggressive antisocial behaviors?

## Materials and Methods

### Participants

The present study analyzed data from the Development of Aggressive Antisocial Behavior Study (DAABS). The DAABS collected baseline data from March 2010 to July 2012 (for in-depth descriptions of the study, see Wallinius et al., 2016; Billstedt et al., 2017; Hofvander et al., 2017). Participants in the DAABS were young (18–25 years, *M* = 22.3, *SD* = 1.9) violent offenders, recruited from nine correctional facilities in the western region of the Swedish Prison and Probation Service. In total, 269 young violent offenders participated in the DAABS, with a participation rate of 71%. The DAABS cohort has been assessed as nationally representative for young violent offenders in Sweden.

### Procedures

All participants who provided informed consent underwent a full-day clinical investigation according to a structured investigation protocol, including file reviews, clinical diagnostic assessments, self-reports, neuropsychological tests, and DNA saliva tests. All investigations were conducted by licensed psychologists with special training in the specific assessments.

### Measures

#### Psychopathic Traits

The Psychopathy Checklist-Revised (PCL-R; Hare, 2003) is a 20-item clinical rating scale that uses interview and collateral information to measure the lifetime prevalence of psychopathic traits. The items are rated on a three-point scale (0 = not present, 1 = present to some degree, 2 = definitely present), with a maximum total score of 40 points. The current study used the four-facet model supported by Neumann et al. (2013) – Interpersonal (i.e., glibness/superficial charm, grandiose sense of self-worth, pathological lying, cunning/manipulative); Affective (i.e., lack of remorse or guilt, shallow affect, callous/lack of empathy, failure to accept responsibility for own actions); Lifestyle (i.e., need for stimulation/proneness to boredom, parasitic lifestyle, lack of realistic long-term goals, impulsivity, irresponsibility); and Antisocial (i.e., poor behavior controls, early behavior problems, juvenile delinquency, revocation of conditional release, criminal versatility) – and the PCL-R total score. In the DAABS cohort, the prevalence of psychopathy was between 15.2% (cutoff = 25) and 4.2% (cutoff = 30). Descriptive data on the PCL-R for the DAABS cohort are provided in Table 1. Complete PCL-R data were available for 262 offenders.

**Table 1:** PCL-R facet and total scores in young violent offenders (*N* = 262).

Variables	Min	Max	*M*	*SD*
PCL-R Interpersonal facet	0.00	8.00	0.98	1.41
PCL-R Affective facet	0.00	8.00	3.23	2.27
PCL-R Lifestyle facet	0.00	10.00	6.44	2.59
PCL-R Antisocial facet	0.00	10.00	6.31	2.86
PCL-R total score	0.00	40.00	17.72	6.94

*SD, standard deviation; PCL-R, Psychopathy Checklist-Revised*.

#### Aggression

The Life History of Aggression (LHA; Brown et al., 1982), a questionnaire originally developed for research on neurobiological correlates to aggression, was used as a measure of lifetime aggressive antisocial behaviors. In the LHA, the frequency of 11 different types of aggressive antisocial behaviors is rated on a five-point scale, based on the number of occurrences since adolescence (0 = no events; 5 = so many events that they cannot be counted), rendering a maximum total score of 55. The LHA was administered as a clinician-rated instrument. The assessor based the ratings on all available information from interviews and files. If the offender reported more aggressive antisocial behaviors than noted in the files, the information from the interviews considered credible by the assessor was used for the analyses. To ensure inter-rater reliability, final LHA scores were assigned through consensus between the assessor and a senior clinician and researcher with considerable experience from the field. Three subscales are defined in the LHA: Aggression, Self-Directed Aggression, and Antisocial Behavior (Coccaro et al., 1997). The Aggression subscale includes items that measure temper tantrums, physical fights, verbal aggression, physical assaults on people or animals, and assaults on property; the Self-Directed Aggression subscale includes items on self-injurious behavior and suicide attempts; and the Antisocial Behavior subscale contains items that describe school disciplinary problems, problems with supervisors at work, and antisocial behavior with or without police involvement. An LHA total score, providing a lifetime history of aggressive antisocial behaviors, is provided by summing the three subscales. In the current study, the scale Self-Directed Aggression was omitted from subscale analyses due to its focus on behaviors that cannot be defined as purely aggressive antisocial. Complete LHA data were available for 267 offenders. Descriptive statistics for the LHA assessments in the DAABS cohort can be retrieved from Wallinius et al. (2016).

#### Intelligence

The Wechsler Adult Intelligence Scale – Third Edition (WAIS-III; Wechsler, 2002) was used to measure intellectual functioning. In this study, the WAIS-III General Ability Index (GAI; Tulsky et al., 2001) was calculated. The GAI is an alternate measure of global intellectual ability comprising six subtests that constitute a Verbal Comprehension Index (VCI; subtests: Information, Similarities, Vocabulary) and a Perceptual Organization Index (POI; subtests: Block design, Matrix reasoning, Picture completion). Complete WAIS-III data were available for 264 offenders.

### Data Analysis Plan

Data were anonymized, coded, and analyzed with IBM SPSS Statistics 22. Since data were found to be non-normally distributed and the normality Kolmogorov–Smirnov statistic significant, non-parametric statistics were used when appropriate. Spearman’s rho was used to test bivariate correlations between psychopathic traits and intelligence and between psychopathic traits and aggressive antisocial behaviors. Simple ordinary least squares (OLS) regressions were used to determine whether intelligence (WAIS-III GAI) moderated the relationship between the four PCL-R facets and aggressive antisocial behaviors (LHA total score) in separate analyses for each facet. Prior to analysis, residuals were checked and determined to be normally distributed. In order to decrease multicollinearity within the models, all independent variables were centered prior to analysis and creation of the moderator variable, in accordance with Iacobucci et al. (2016). Centering rather than standardization was chosen since this retains the possibility to interpret the regression coefficients as usual. For all models, variance inflation factor and tolerance values were acceptable, indicating that multicollinearity was not an issue in the models. Since the scores on the Interpersonal facet were highly skewed (see Table 1), we calculated two different regression models for the Interpersonal facet, where model 1 was similar to the other facets, and model 2 included two extra independent variables: (1) a dichotomized variable for the Interpersonal facet (0 = 0 score, 1 = score > 0), and (2) a moderator variable with the dichotomized Interpersonal variable X the GAI scores. This was executed to separate the effects of any kind of interpersonal psychopathic traits (the dichotomized variable) and the degree of interpersonal psychopathic traits (the original Interpersonal facet score), and whether this affected the moderation. However, it should be noted that model 2 produced somewhat higher multicollinearity within the model, yet within the acceptable range.

### Ethics Statement

The study was approved by the Regional Ethics Review Board in Lund, Dnr 2009/405, as part of the DAABS project, and executed in accordance with the Declaration of Helsinki. Participation was voluntary, and all participants provided written informed consent before participation. After completing participation, each participant received 200 SEK as compensation for the time they spent in the study instead of working in the Swedish Prison and Probation Service. Participants who demonstrated current mental illness were offered referral to prison doctors, and psychiatrists if available, for further assessments.

## Results

Psychopathic traits were positively associated with aggressive antisocial behaviors for all facets except for the Interpersonal facet (Table 2). The Affective facet was negatively correlated to both the WAIS GAI (*r_s_* = -0.16, *P* = 0.010) and the WAIS POI (*r_s_* = -0.15, *P* = 0.018), while no significant correlations to the WAIS VCI were found for any of the PCL-R facets.

**Table 2:** Associations (Spearman’s rho) between psychopathic traits (PCL-R) and aggressive antisocial behaviors (LHA).

	LHA	LHA	LHA	WAIS-	WAIS-	WAIS-	Statistic
	Total score	Aggression	Antisocial	III GAI	III VCI	III POI	
			behavior				
PCL-R Interpersonal facet	0.059	0.037	0.091	0.040	0.008	0.078	*r_s_*
	0.338	0.546	0.140	0.517	0.895	0.213	*P*
PCL-R Affective facet	**0.184**	**0.183**	**0.190**	**-160**	-0.113	**-0.147**	*r_s_*
	**0.003**	**0.003**	**0.002**	**0.010**	0.069	**0.018**	*P*
PCL-R Lifestyle facet	**0.466**	**0.398**	**0.495**	-0.054	-0.017	-0.080	*r_s_*
	**0.000**	**0.000**	**0.000**	0.383	0.781	0.198	*P*
PCL-R Antisocial facet	**0.506**	**0.453**	**0.526**	0.021	0.009	0.010	*r_s_*
	**0.000**	**0.000**	**0.000**	0.736	0.890	0.879	*P*
PCL-R total score	**0.473**	**0.419**	**0.493**	-0.054	-0.035	-0.058	*r_s_*
	**0.000**	**0.000**	**0.000**	0.387	0.573	0.354	*P*

*Significant correlations between psychopathic traits (PCL-R) and aggressive antisocial behaviors (LHA) are shown in bold. LHA, Life History of Aggression*.

**Figure 1:**
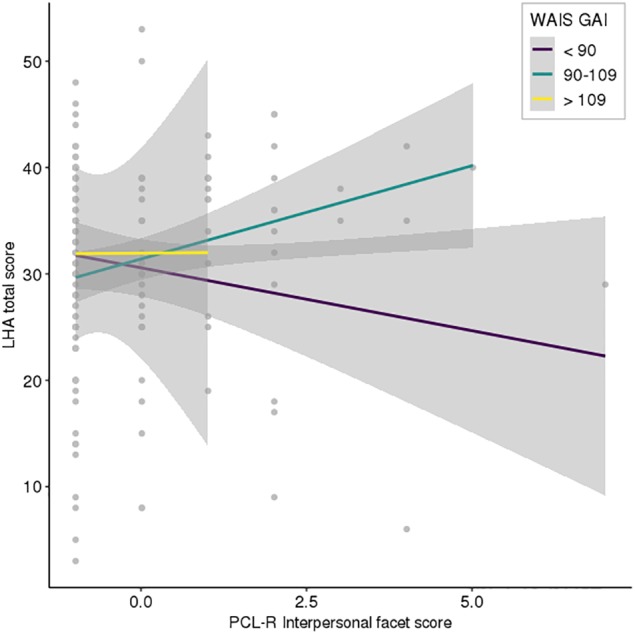
Interaction between Interpersonal psychopathic traits and levels of intelligence in the prediction of aggressive antisocial behaviors. The plotted values for the Psychopathy Checklist-Revised (PCL-R) Interpersonal facet and Wechsler Adult Intelligence Scale – Third Edition (WAIS-III) General Ability Index (GAI) scores are, in accordance with the regression analysis, centered why negative values are possible.

In the moderation analyses (Table 3), the only model demonstrating a statistically significant moderation effect was for the Interpersonal facet, where WAIS GAI scores moderated the effect of the Interpersonal facet on the LHA total scores in both models 1 and 2. However, neither the Interpersonal facet nor the WAIS GAI scores were independent predictors of LHA total scores in the model. Including the dichotomized Interpersonal variable in model 2 did not provide a significant independent or moderation effect. The amount of variance explained by the models for the Interpersonal facet was very small (see Table 3). Also, as demonstrated in Figure 1, there were outliers affecting the results and the 95% CIs were large. For the other models, the Affective, Lifestyle, and Antisocial facets were statistically significant predictors of the LHA total score. Furthermore, in the model testing the Lifestyle facet, the WAIS GAI was a statistically significant predictor, albeit with no moderation effect. The models testing the Lifestyle and Antisocial facets had the highest amount of explained variance in LHA total scores (see Table 3).

**Table 3:** Simple ordinary least squares (OLS) regressions, testing independent and moderation effects of psychopathic traits (PCL-R) and intelligence (WAIS-III GAI) in the prediction of aggressive antisocial behaviors (LHA total score).

	β	*P*	95.0% CI		*r*2
**PCL-R Interpersonal facet**				
**Model 1**				
Interpersonal facet	-0.009	0.887	-0.946	0.819	0.028
WAIS-III GAI	0.101	0.107	-0.020	0.207	
Interpersonal facet X WAIS-III GAI	0.147	0.020	0.017	0.193	
**Model 2**				
Interpersonal facet	-0.096	0.312	-2.035	0.653	0.038
Interpersonal facet (dichotomized)	0.111	0.242	-1.531	6.034	
WAIS-III GAI	0.212	0.061	-0.010	0.402	
Interpersonal facet X WAIS-III GAI	0.244	0.019	0.028	0.319	
Interpersonal facet (dichotomized) X WAIS-III GAI	-0.151	0.256	-0.602	0.161	
**PCL-R Affective facet**				
Affective facet	0.216	0.001	0.422	1.512	0.052
WAIS-III GAI	0.114	0.069	-0.008	0.218	
Affective facet X WAIS-III GAI	-0.003	0.965	-0.053	0.051	
**PCL-R Lifestyle facet**				
Lifestyle facet	0.561	0.000	1.803	2.603	0.322
WAIS-III GAI	0.108	0.040	0.004	0.195	
Lifestyle facet X WAIS-III GAI	-0.071	0.173	-0.062	0.011	
**PCL-R Antisocial facet**				
Antisocial facet	0.582	0.000	1.714	2.425	0.345
WAIS-III GAI	0.068	0.182	-0.030	0.156	
Antisocial facet X WAIS-III GAI	-0.029	0.572	-0.041	0.023	

*The β-values provided in the table are standardized. WAIS-III GAI, Wechsler Adult Intelligence Scale – Third Edition, General Ability Index*.

## Discussion

The present study investigated whether intelligence, which is generally important for rehabilitation strategies and everyday functioning in forensic contexts, is relevant in the association between psychopathic traits and aggressive antisocial behaviors. The aims were to identify associations between the four facets of psychopathy according to the PCL-R, aggressive antisocial behaviors according to the LHA, and intelligence as measured by the WAIS-III GAI, and to test whether intelligence moderated the associations between psychopathic traits and aggressive antisocial behaviors. Our analyses yielded two main findings. First, our results indicated differing positive associations (weak to strong) between psychopathic traits and aggressive antisocial behaviors in young violent offenders depending on facet examined, with the strongest associations with the Lifestyle and Antisocial facets and the PCL-R total score and no significant associations to the Interpersonal facet scores. Only the Affective facet was weakly, negatively correlated to measures of intelligence. In the moderation analyses, a small yet statistically significant moderation effect of intelligence on the association between the Interpersonal facet and LHA total scores was demonstrated. However, the amount of variance in the LHA total scores explained by the model was very small: 2.9% (see Table 3).

The results from the moderation analyses, with a small but statistically significant moderation effect of WAIS-III GAI on the relation between Interpersonal facet scores and LHA total scores, are interesting but must be interpreted with caution. First, the Interpersonal facet scores were highly skewed in our sample of young violent offenders, with the majority of the participants scoring “0” (see Figure 1). That is, even though our results might seem interesting and possibly in line with Cleckley’s original descriptions of interpersonal psychopathic characteristics including “good intelligence,” the interpretation is affected by the low variation in Interpersonal facet scores. Through our inclusion of a second model, including a dichotomized Interpersonal facet variable, we tried to separate the effects of any kind of interpersonal psychopathic traits (the dichotomized variable) and the degree of interpersonal psychopathic traits in the moderation analysis. Here, our results can be interpreted as the moderation effect being affected not by the mere presence of interpersonal psychopathic traits, but rather on the degree of interpersonal psychopathic traits. In Figure 1, the participants with an intelligence quotient within the average range (IQ 90–109) demonstrated increased aggressive antisocial behaviors with higher scores on the Interpersonal facet, while participants with an intelligence quotient below average or in the lower average range (IQ < 90) demonstrated an inverse relationship between Interpersonal facet scores and LHA total scores. Still, our findings must be considered exploratory, and replications are needed before conclusions can be drawn. Furthermore, the variance in aggressive antisocial behaviors (LHA total scores) explained by the model was very low, even within behavioral sciences.

The results of the moderation analyses for the other PCL-R facets were in line with what could be expected from the results already described, with intelligence not being a significant moderator of the association between psychopathy and aggressive antisocial behaviors. However, WAIS-III GAI scores were a statistically significant, independent predictor in the multivariate regression analysis. When compared to the correlations reported in Table 2, this seems to be an argument for the cautious interpretation of *p*-values rather than as evidence of an effect that holds clinical value. In summary, we could not confirm the findings of Hampton et al. (2014) in our sample. Since, to the best of our knowledge, only two studies have been published on this specific subject, it is premature to draw any firm conclusions. Also, the discrepancies between our findings and those of Hampton et al. (2014) may be affected by differences in study designs. The study by Hampton et al. (2014) was prospective, following over 1,000 juvenile delinquents aged 14–17 with a variety of index offenses, and in the follow-up used only self-reported offenses. Our study was cross-sectional, included only violent offenders in young adulthood, applied a somewhat broader measure of intellectual functioning, and combined self-reported offenses with information on offenses drawn from files.

Our findings of associations between psychopathic traits and aggressive antisocial behaviors are in line with previous research demonstrating strong relations between these concepts (Grann et al., 1999; Hare, 1999; Gustavson et al., 2007; Hare and Neumann, 2008; Sánchez et al., 2014; Harris et al., 2017). However, it is clear that the associations differ depending on which facet is investigated. For instance, the Interpersonal facet was not significantly correlated to any aggressive antisocial behaviors investigated in this study. This is in line with previous research demonstrating unique properties for the Interpersonal facet in relation both to clinical concepts on the externalizing spectrum and to aggressive antisocial behaviors (e.g., Wallinius et al., 2012) and calls for caution in using the Interpersonal facet as a part of violence risk assessments. It is, however, possible that the types of aggressive antisocial behaviors measured by the LHA are not relevant to interpersonal characteristics depicted in the Interpersonal facet, since previous research has identified an association between instrumental violence and the Interpersonal facet (Walsh et al., 2009). The LHA was not designed specifically to measure instrumental violence; instead, it assesses aggressive antisocial behaviors that are more likely to be reactive rather than instrumental. Thus, it was not possible to investigate a possible association between intelligence and instrumental aggressive antisocial behaviors in the current study, but that could be a focus of future studies.

We found a weak, positive correlation between the Affective facet and aggressive antisocial behaviors, in line with previous findings from Sreenivasan et al. (2008) where the Affective facet was related to the number of violent acts. However, it should be noted that our sample and the Sreenivasan sample differed in that the Sreenivasan sample had much higher levels of psychopathic traits than our sample. Our study adds evidence, however, to the growing acknowledgment that – despite the overall destructiveness of the personality traits assembled in them – the Interpersonal and Affective facets of psychopathy might not be as relevant to (reactive) aggressive antisocial behaviors as may be assumed. The Lifestyle and Antisocial facets were, however, as implied by the behaviors constituting them, associated moderately to strongly (0.40 ≥ *r*_s_ ≤ 0.53) with aggressive antisocial behaviors, with a pattern of somewhat stronger correlations to antisocial than to aggressive behaviors. The results of a moderate-to-strong correlation between the PCL-R total score and aggressive antisocial behaviors should be interpreted in light of the associations with the Lifestyle and Antisocial facets, and the fact that behaviors in these facets were more common in the group than the behaviors in the Interpersonal and Affective facets (see Table 1).

In our investigations into associations between the PCL-R four-facet model and the applied WAIS-III measures, only the Affective facet (i.e., lack of remorse or guilt, shallow affect, callous/lack of empathy, failure to accept responsibility for own actions) was significantly, negatively, albeit weakly, correlated to both general and perceptual dimensions of intelligence. Due to the weak correlation, interpretations should be made with caution. Yet, the results are in line with previous findings by Salekin et al. (2004), demonstrating a negative relationship between affective, psychopathic traits, and intelligence in juvenile offenders. Our results could also be seen in light of the general disabilities associated with lower intelligence, such as problems with taking another’s perspective (Decety et al., 2013) and deficits in theory of mind (Sandvik et al., 2014). Previous research has demonstrated that individuals high in psychopathic traits can deliberately take the perspective of others but show cognitive deficits in forming automatic representations of others’ perspectives (Drayton et al., 2018). These deficits have been associated with aggressive antisocial behaviors suggested to result from a focus on goal-driven perspectives that indicate an attention deficit in psychopathic individuals. Could it be that our results could be indicative of a cognitive immaturity that reflects in seemingly shallow affects, particularly noticeable in younger offenders?

It should also be noted that we found no associations between the PCL-R facets and the verbal dimensions of intelligence in our study. As recently pointed out by Walters and Duncan (2018), previous findings of associations between PCL-R facets and intelligence have been disparate and have applied varying measures of (verbal) intelligence. The study by Walters et al. (2018) is the most comparable to our study, and it demonstrated that all facets, except Interpersonal, were weakly to moderately negatively correlated to measures of (particularly verbal) intelligence. Even though persons with highly psychopathic traits might seem to demonstrate a verbal intelligence above average at first glance, this may be misleading and, when investigated thoroughly, poorly executed and absurd and affected by cognitive deficits and low education (DeLisi et al., 2010; DeLisi, 2016). Thus, this needs to be considered in designing studies on this matter, e.g., in choosing adequate measures of verbal intelligence that may be less affected by such strategies.

Unfortunately, the research evidence of an association between psychopathic traits and intelligence is still ambiguous and hampered by differences in study designs. That said, self-control (operationalized as self-directedness) and tolerance of others (operationalized as cooperativeness) have been demonstrated to be protective against aggression and antisocial behaviors (Kerekes et al., 2017). Self-directedness and cooperativeness are character traits that are part of a person’s mental self-government (Cloninger, 2004) and are, in fact, almost the opposite of psychopathic behaviors (e.g., Garcia and Rosenberg, 2016). In other words, a person who is both highly self-directed and cooperative is responsible, self-acceptant, helpful, empathic, and socially tolerant (Cloninger, 2004). Interestingly, as with psychopathy in this study, self-directedness and cooperativeness have not been associated with intelligence (Mousavi et al., 2015). In future studies, investigations of the importance of general personality traits, such as self-directedness and cooperativeness, might provide increased knowledge on moderators of the association between psychopathy and aggression.

Based on the, so far, contradictory findings about whether intelligence is a moderator of the association between different facets of psychopathy and aggressive antisocial behaviors, we suggest replications of our work and further studies on this subject. Continued research needs to consider the different contributions of the PCL-R facets, and also to expand the investigations to other samples, especially samples where higher scores on the Interpersonal facet can be expected. In this, researchers need to choose measures with care, and preferably use different measures of the same condition, e.g., psychopathy, intelligence, and aggressive antisocial behaviors, to provide a clearer picture of the constructs of interest (Sharratt et al., 2019; Wallinius et al., 2019). It is crucial to find methods to reduce aggression in violent offenders such as those in our sample, and the need for continued research in this field is vast in order to design and deliver effective treatment interventions. Thus, a continued search for plausible moderators or mediators of the relation between psychopathic traits and aggressive antisocial behaviors is called for.

### Limitations

This study is limited by its cross-sectional design. However, the current sample was large and nationally representative for Sweden, and the data were derived from thorough clinical investigations considering not only self-reports but also official information on aggressive antisocial behaviors. Based on previous findings, investigations into whether different patterns may be distinguished between groups with low-to-moderate versus highly psychopathic traits could be recommended. This, however, was not possible in this study due to the low prevalence of highly psychopathic traits in our sample, which might be explained by the relative youth of the participating offenders whose psychopathic traits might not yet have been fully established. Another possible explanation is maybe because offenders in European settings generally score lower on the PCL-R compared to their North American counterparts (Cooke et al., 2005). A further important limitation in our study was the lack of female offenders in the sample; therefore, the results are not generalizable to female populations. However, although generalizations should be made with care and applied only to similar groups, previous studies on the DAABS cohort (Billstedt et al., 2017; Hofvander et al., 2017) have demonstrated a highly elevated prevalence of mental disorders among the participants, which suggests that investigations of this group should also be relevant to other forensic contexts, such as forensic psychiatry.

## Conclusion

The present study examined the association between psychopathic traits, aggressive antisocial behaviors, and intelligence in young violent offenders and tested whether intelligence moderates the presumed relationship between psychopathy and aggressive antisocial behaviors. We demonstrated that both bivariate and multivariate associations and moderation effects varied depending on which facet that was investigated, and that the Interpersonal and Affective facets had very low impact on the explained variance in aggressive antisocial behaviors in young violent offenders. We suggest that intelligence, although important for rehabilitation strategies and everyday functioning, is not necessarily pertinent to understand aggressive antisocial or psychopathic behaviors in young violent offenders.

## Ethics Statement

This study was carried out in accordance with the recommendations of the Regional Ethics Review Board in Lund, with written informed consent from all subjects. All subjects gave written informed consent in accordance with the Declaration of Helsinki. The protocol was approved by the Regional Ethics Review Board in Lund, Dnr 2009/405.

## Author Contributions

FGM, MW, and DG contributed to the conception and design of the study. EB collected the data. FGM, MW, and DG performed the statistical analysis. FGM wrote the first draft of the manuscript. All authors wrote the sections of the manuscript and contributed to manuscript revision, read, and approved the submitted version.

## Conflict of Interest Statement

The authors declare that the research was conducted in the absence of any commercial or financial relationships that could be construed as a potential conflict of interest.

## References

[B1] Babiak P. Hare R. D. (2006). *Snakes in Suits: When Psychopaths Go to Work*. New York, NY: Regan Books.

[B2] Beaver K. M. Schwartz J. A. Nedelec J. L. Connolly E. J. Boutwell B. B. Barnes J. C. (2013). Intelligence is associated with criminal justice processing: arrest through incarceration. *Intelligence* 41 277–288. 10.1016/j.intell.2013.05.001

[B3] Billstedt E. Anckarsäter H. Wallinius M. Hofvander B. (2017). Neurodevelopmental disorders in young violent offenders: overlap and background characteristics. *Psychiatry Res.* 252 234–241. 10.1016/j.psychres.2017.03.004 28285251

[B4] Boduszek D. Debowska A. Dhingra K. DeLisi M. (2016). Introduction and validation of psychopathic personality traits scale (PPTS) in a large prison sample. *J. Crim. Justice* 46 9–17. 10.1016/j.jcrimjus.2016.02.004

[B5] Brown G. L. Ebert M. H. Goyer P. F. Jimerson D. C. Klein W. J. Bunney W. E. Jr. (1982). Aggression, suicide, and serotonin: relationships to CSF amine metabolites. *Am. J. Psychiatry* 139 741–746. 10.1176/ajp.139.6.741 6177256

[B6] Cleckley H. (1950). *The Mask of Sanity*, 2nd Edn St Louis, MO: Mosby.

[B7] Cloninger C. R. (2004). *Feeling Good: The Science of Well-Being*. New York, NY: Oxford University Press.

[B8] Coccaro E. F. Berman M. E. Kavoussi R. J. (1997). Assessment of life history of aggression: development and psychometric characteristics. *Psychiatry Res.* 73 147–157. 10.1016/s0165-1781(97)00119-4 9481806

[B9] Coid J. Yang M. Ullrich S. Roberts A. Hare R. D. (2009a). Prevalence and correlates of psychopathic traits in the household population of Great Britain. *Int. J. Law Psychiatry* 32 65–73. 10.1016/j.ijlp.2009.01.002 19243821

[B10] Coid J. Yang M. Ullrich S. Roberts A. Moran P. Bebbington P. (2009b). Psychopathy among prisoners in England and Wales. *Int. J. Law Psychiatry* 32 134–141. 10.1016/j.ijlp.2009.02.008 19345418

[B11] Cooke D. J. Michie C. Hart S. D. Clark D. (2005). Assessing psychopathy in the UK: concerns about cross-cultural generalisability. *Br. J. Psychiatry* 186 335–341. 10.1192/bjp.186.4.335 15802692

[B12] Cornell D. G. Warren J. Hawk G. Stafford E. Oram G. Pine D. (1996). Psychopathy in instrumental and reactive violent offenders. *J. Consult. Clin. Psychol.* 64 783–790. 10.1037//0022-006x.64.4.783 8803369

[B13] Debowska A. Boduszek D. Dhingra K. Sherretts N. Willmott D. DeLisi M. (2018). Can we use Hare’s psychopathy model within forensic and non-forensic populations? An empirical investigation. *Deviant Behav.* 39 224–242. 10.1080/01639625.2016.1266887

[B14] Decety J. Chen C. Harenski C. Kiehl K. A. (2013). An fMRI study of affective perspective taking in individuals with psychopathy: imagining another in pain does not evoke empathy. *Front. Hum. Neurosci.* 7:489. 10.3389/fnhum.2013.00489 24093010 PMC3782696

[B15] DeLisi M. (2016). *Palgrave’s Frontiers in Criminology Theory. Psychopathy as Unified Theory of Crime*. New York, NY: Palgrave Macmillan, 10.1057/978-1-137-46907-6

[B16] DeLisi M. Reidy D. E. Heirigs M. H. Tostlebe J. J. Vaughn M. G. (2017). Psychopathic costs: a monetization study of the fiscal toll of psychopathy features among institutionalized delinquents. *J. Crim. Psychol.* 8 112–124. 10.1108/JCP-07-2017-0031

[B17] DeLisi M. Vaughn M. G. Beaver K. M. Wright J. P. (2010). The Hannibal Lecter myth: psychopathy and verbal intelligence in the MacArthur violence risk assessment study. *J. Psychopathol. Behav. Assess.* 32 169–177. 10.1007/s10862-009-9147-z

[B18] Dhingra K. Boduszek D. (2013). Psychopathy and criminal behaviour: a psychosocial research perspective. *J. Crim. Psychol.* 3 83–107. 10.1108/jcp-06-2013-0014

[B19] Drayton L. A. Santos L. R. Baskin-Sommers A. (2018). Psychopaths fail to automatically take the perspective of others. *Proc. Natl. Acad. Sci. U.S.A.* 115 3302–3307. 10.1073/pnas.1721903115 29531085 PMC5879707

[B20] Garcia D. Rosenberg P. (2016). The dark cube: dark and light character profiles. *PeerJ.* 4:e1675. 10.7717/peerj.1675 26966650 PMC4783766

[B21] Grann M. Långström N. Tengström A. Kullgren G. (1999). Psychopathy (PCL-R) predicts violent recidivism among criminal offenders with personality disorders in Sweden. *Law Hum. Behav.* 23 205–217. 10.1023/a:1022372902241 10333757

[B22] Gustavson C. Ståhlberg O. Sjödin A. K. Forsman A. Nilsson T. Anckarsäter H. (2007). Age at onset of substance abuse: a crucial covariate of psychopathic traits and aggression in adult offenders. *Psychiatry Res.* 153 195–198. 10.1016/j.psychres.2006.12.020 17659353

[B23] Hampton A. S. Drabick D. A. Steinberg L. (2014). Does IQ moderate the relation between psychopathy and juvenile offending? *Law Hum. Behav.* 38 23–33. 10.1037/lhb0000036 23750597 PMC3864133

[B24] Hare R. D. (1985). Comparison of procedures for the assessment of psychopathy. *J. Consult. Clin. Psychol.* 53 7–16. 10.1037/0022-006X.53.1.7 3980831

[B25] Hare R. D. (1996). Psychopathy: a clinical construct whose time has come. *Crim. Justice Behav.* 23 25–54.

[B26] Hare R. D. (1999). *Without Conscience: The Disturbing World of the Psychopaths Among Us*. New York, NY: Guilford Press.

[B27] Hare R. D. (2003). *Hare Psychopathy Checklist-Revised Technical Manual*, 2nd Edn Toronto, CA: Multi-Health Systems.

[B28] Hare R. D. Neumann C. S. (2008). Psychopathy as a clinical and empirical construct. *Annu. Rev. Clin. Psychol.* 4 217–246. 10.1146/annurev.clinpsy.3.022806.091452 18370617

[B29] Hare R. D. Neumann C. S. (2010). The role of antisociality in the psychopathy construct: comment on Skeem and Cooke (2010). *Psychol. Assess.* 22 446–454. 10.1037/a0013635 20528070

[B30] Hare R. D. Strachan C. E. Forth A. E. (1993). “Psychopathy and crime: a review,” in *Clinical Approaches to the Mentally Disordered Offender*, eds Howells K. Hollin C. R. (Chichester: Wiley), 165–178.

[B31] Harris P. B. Boccaccini M. T. Rice A. K. (2017). Field measures of psychopathy and sexual deviance as predictors of recidivism among sexual offenders. *Psychol. Assess.* 29 639–651. 10.1037/pas0000394 28594208

[B32] Hart S. D. Kropp P. R. Hare R. D. (1988). Performance of male psychopaths following conditional release from prison. *J. Consult. Clin. Psychol.* 56 227–232. 3372830 10.1037//0022-006x.56.2.227

[B33] Hecht L. K. Latzman R. D. Lilienfeld S. O. (2018). “The psychological treatment of psychopathy,” in *Evidence-Based Psychotherapy: The State of the Science and Practice*, eds David D. Lynn S. Jay Montgomery G. H. (Hoboken, NJ: Wiley-Blackwell), 271–298.

[B34] Hofvander B. Anckarsäter H. Wallinius M. Billstedt E. (2017). Mental health among young adults in prison: the importance of childhood-onset conduct disorder. *Br. J. Psychiatry Open* 3 78–84. 10.1192/bjpo.bp.116.003889 28357134 PMC5362727

[B35] Iacobucci D. Schneider M. J. Popovich D. L. Bakamitsos G. A. (2016). Mean centering helps alleviate “micro” but not “macro” multicollinearity. *Behav. Res. Methods* 48 1308–1317. 26148824 10.3758/s13428-015-0624-x

[B36] Johansson P. Kerr M. (2005). Psychopathy and intelligence: a second look. *J. Pers. Disord.* 19 357–369. 16178679 10.1521/pedi.2005.19.4.357

[B37] Kavish N. Bailey C. Sharp C. Venta A. (2018). On the relation between general intelligence and psychopathic traits: an examination of inpatient adolescents. *Child Psychiatry Hum. Dev.* 49 341–351. 10.1007/s10578-017-0754-8 28836093

[B38] Kerekes N. Falk Ö Brändström S. Anckarsäter H. Råstam M. Hofvander B. (2017). The protective effect of character maturity in child aggressive antisocial behavior. *Compr. Psychiatry* 76 129–137. 10.1016/j.comppsych.2017.04.007 28521251

[B39] Kosson D. S. Smith S. S. Newman J. P. (1990). Evaluating the construct validity of psychopathy in Black and White male inmates: three preliminary studies. *J. Abnorm. Psychol.* 99 250–259. 2212275 10.1037//0021-843x.99.3.250

[B40] Lilienfeld S. O. Smith S. F. Sauvigné K. C. Patrick C. J. Drislane L. E. Latzman R. D. (2016). Is boldness relevant to psychopathic personality? Meta-analytic relations with non-Psychopathy Checklist-based measures of psychopathy. *Psychol. Assess.* 28 1172–1185. 26619088 10.1037/pas0000244

[B41] Logan M. Hare R. D. (2008). “Criminal psychopathy: an introduction for police,” in *Psychology of Criminal Investigation* eds St-Yves M. Tanguay M. (Cowansville, QC: Editions Yvon Blais), 393–442.

[B42] Mousavi F. Rozsa S. Nilsson T. Archer T. Anckarsäter H. Garcia D. (2015). Personality and intelligence: persistence, not self-directedness, cooperativeness or self-transcendence, is related to twins’ cognitive abilities. *PeerJ.* 3:e1195. 10.7717/peerj.1195 26312186 PMC4548492

[B43] Neumann C. S. Hare R. D. (2008). Psychopathic traits in a large community sample: links to violence, alcohol use, and intelligence. *J. Consult. Clin. Psychol.* 76 893–899. 10.1037/0022-006X.76.5.893 18837606

[B44] Neumann C. S. Hare R. D. Pardini D. A. (2015). Antisociality and the construct of psychopathy: data from across the globe. *J. Pers.* 83 678–692. 10.1111/jopy.12127 25181550

[B45] Neumann C. S. Johansson P. T. Hare R. D. (2013). The Psychopathy Checklist-Revised (PCL-R), low anxiety, and fearlessness: a structural equation modeling analysis. *Pers. Disord.* 4 129–137. 10.1037/a0027886 22642462

[B46] Pardini D. A. Raine A. Erickson K. Loeber R. (2014). Lower amygdala volume in men is associated with childhood aggression, early psychopathic traits, and future violence. *Biol. Psychiatry* 75 73–80. 10.1016/j.biopsych.2013.04.003 23647988 PMC3751993

[B47] Pedersen L. Kunz C. Rasmussen K. Elsass P. (2010). Psychopathy as a risk factor for violent recidivism: investigating the psychopathy checklist screening version (PCL: SV) and the comprehensive assessment of psychopathic personality (CAPP) in a forensic psychiatric setting. *Int. J. Forensic Ment. Health* 9 308–315.

[B48] Raine A. Dodge K. Loeber R. Gatzke-Kopp L. Lynam D. Reynolds C. (2006). The reactive–proactive aggression questionnaire: differential correlates of reactive and proactive aggression in adolescent boys. *Aggress. Behav.* 32 159–171. 20798781 10.1002/ab.20115 PMC2927832

[B49] Reidy D. E. Shelley-Tremblay J. F. Lilienfeld S. O. (2011). Psychopathy, reactive aggression, and precarious proclamations: a review of behavioral, cognitive, and biological research. *Aggress. Violent Behav.* 16 512–524. 10.1016/j.avb.2011.06.002

[B50] Rice M. E. Harris G. T. (2013). “Psychopathy and violent recidivism,” in *Oxford Series in Neuroscience, Law, and Philosophy. Handbook on Psychopathy and Law*, eds Kiehl K. A. Sinnott-Armstrong W. P. (New York, NY: Oxford University Press), 231–249.

[B51] Salekin R. T. Neumann C. S. Leistico A. M. R. Zalot A. A. (2004). Psychopathy in youth and intelligence: an investigation of Cleckley’s hypothesis. *J. Clin. Child Adolesc. Psychol.* 33 731–742. 15498740 10.1207/s15374424jccp3304_8

[B52] Sánchez J. C. Vergara R. G. Moraga F. R. G. Castro R. N. (2014). Psicopatía y delincuencia: comparaciones y diferencias entre ofensores sexuales y delincuentes comunes en una cárcel chilena. *Criminalidad* 56 229–245. 22849945

[B53] Sandvik A. M. Hansen A. L. Johnsen B. H. Laberg J. C. (2014). Psychopathy and the ability to read the “language of the eyes”: divergence in the psychopathy construct. *Scand. J. Psychol.* 55 585–592. 10.1111/sjop.12138 24954681 PMC4282377

[B54] Schwartz J. A. Savolainen J. Aaltonen M. Merikukka M. Paananen R. Gissler M. (2015). Intelligence and criminal behavior in a total birth cohort: an examination of functional form, dimensions of intelligence, and the nature of offending. *Intelligence* 51 109–118.

[B55] Sharratt K. Boduszek D. Retzler C. (2019). Clarifying the relationship between psychopathy and intelligence using four dimensions of the WASI-II. *Deviant Behav.* 10.1080/01639625.2019.1582968

[B56] Shaw J. Porter S. (2012). “Forever a psychopath? Psychopathy and the criminal career trajectory,” in *Psychopathy and Law: A Practitioner’s Guide*, eds Nyholm J.-O. äkkänen-Nyholm H. H. (Hoboken, NY: John Wiley & Sons), 201–221.

[B57] Skeem J. L. Cooke D. J. (2010a). Is criminal behavior a central component of psychopathy?. Conceptual directions for resolving the debate. *Psychol. Assess.* 22 433–445. 10.1037/a0008512 20528069

[B58] Skeem J. L. Cooke D. J. (2010b). One measure does not a construct make: directions toward reinvigorating psychopathy research—reply to Hare and Neumann (2010). *Psychol. Assess.* 22 455–459. 10.1037/a0014862 20528071

[B59] Sreenivasan S. Walker S. C. Weinberger L. E. Kirkish P. Garrick T. (2008). Four-Facet PCL–R structure and cognitive functioning among high violent criminal offenders. *J. Pers. Assess.* 90 197–200. 18444114 10.1080/00223890701845476

[B60] Tulsky D. S. Saklofske D. H. Wilkins C. Weiss L. G. (2001). Development of a general ability index for the Wechsler adult intelligence scale—Third Edition. *Psychol. Assess.* 13 566–571. 11793899 10.1037//1040-3590.13.4.566

[B61] Verschuere B. van Ghesel Grothe S. Waldorp L. Watts A. L. Lilienfeld S. O. Edens J. F. (2018). What features of psychopathy might be central? A network analysis of the psychopathy checklist-revised (PCL-R) in three large samples. *J. Abnorm. Psychol.* 127 51–65. 10.1037/abn0000315 29172600

[B62] Wallinius M. (2012). *Aggressive Antisocial Behavior-Clinical, Cognitive, and Behavioral Covariates of Its Persistence*. Doctoral Dissertation, Lund University, Lund.

[B63] Wallinius M. Delfin C. Billstedt E. Nilsson T. Anckarsäter H. Hofvander B. (2016). Offenders in emerging adulthood: school maladjustment, childhood adversities, and prediction of aggressive antisocial behaviors. *Law Hum. Behav.* 40 551–565. 10.1037/lhb0000202 27243360

[B64] Wallinius M. Nilsson T. Hofvander B. Anckarsäter H. Stålenheim G. (2012). Facets of psychopathy among mentally disordered offenders: clinical comorbidity patterns and prediction of violent and criminal behavior. *Psychiatry Res.* 198 279–284. 10.1016/j.psychres.2012.01.005 22421067

[B65] Wallinius M. Nordholm J. Wagnström F. Billstedt E. (2019). Cognitive functioning and aggressive antisocial behaviors in young violent offenders. *Psychiatry Res.* 272 572–580. 10.1016/j.psychres.2018.12.140 30616126

[B66] Walsh Z. Swogger M. T. Kosson D. S. (2009). Psychopathy and instrumental violence: facet level relationships. *J. Pers. Disord.* 23 416–424. 10.1521/pedi.2009.23.4.416 19663661 PMC3959160

[B67] Walters G. D. Duncan S. A. (2018). Performance-verbal discrepancies and facets of psychopathy: assessing the relationship between WAIS–R/III summary IQs/index scores and PCL–R facet scores. *J. Crim. Psychol.* 8 234–246. 10.1108/JCP-12-2017-0045

[B68] Walters G. D. Knight R. A. Grann M. Dahle K. P. (2008). Incremental validity of the psychopathy checklist facet scores: predicting release outcome in six samples. *J. Abnorm. Psychol.* 117 396–405. 10.1037/0021-843X.117.2.396 18489215

[B69] Walters G. D. Wilson N. J. Glover A. J. (2011). Predicting recidivism with the psychopathy checklist: are factor score composites really necessary? *Psychol. Assess.* 23 552–557. 10.1037/a0022483 21299307

[B70] Wechsler D. (2002). *WAIS-III: Wechsler Adult Intelligence Scale*, 3rd Edn San Antonio, TX: Psychological Corporation.

[B71] Yildirim B. O. Derksen J. J. (2015). Clarifying the heterogeneity in psychopathic samples: towards a new continuum of primary and secondary psychopathy. *Aggress. Violent Behav.* 24 9–41.

